# Effects of Cooking Methods on Caffeoylquinic Acids and Radical Scavenging Activity of Sweet Potato

**DOI:** 10.3390/foods13071101

**Published:** 2024-04-03

**Authors:** Megumi Kido, Makoto Yoshimoto, Kozue Sakao, Koji Wada, De-Xing Hou

**Affiliations:** 1 The United Graduate School of Agricultural Sciences, Kagoshima University, 1-21-24 Korimoto, Kagoshima 890-0065, Japan; kido@jkajyo.ac.jp (M.K.); sakaok24@agri.kagoshima-u.ac.jp (K.S.); kojiwada@agr.u-ryukyu.ac.jp (K.W.); 2 Department of Human Life and Science, Kagoshima Women’s College, 6-9 Kohraicho, Kagoshima 890-8565, Japan; 3 My Food Development Institute, 109-3 Miyakonojo, Miyazaki 885-0041, Japan; mak825@btvm.ne.jp; 4 Faculty of Agricultural Sciences, Kagoshima University, 1-21-24 Korimoto, Kagoshima 890-0065, Japan; 5 Faculty of Agriculture, University of the Ryukyus, Senbaru, Nishihara 1, Okinawa 903-0213, Japan

**Keywords:** cooking, caffeoylquinic acid, radical scavenging activity, sweet potato

## Abstract

The effects of cooking methods, including steaming, deep-frying, and baking, on the phenolic content, 1,1-diphenyl-2-picrylhydrazyl radical scavenging activity, and isomerization of caffeoylquinic acids in sweet potato were investigated. A high correlation was observed between antioxidant capacity and total phenolic content. Deep-frying treatment resulted in higher antioxidant capacity with increasing heating time. The major phenolic components of raw sweet potat were 5-caffeoylquinic acid (CQA) and 3,5-dicaffeoylquinic acid (diCQA), which were reduced by heat treatment due to the isomerization of 5-CAQ to 3- and 4-CQA, and 3,5-diCQA to 3,4- and 4,5-diCQA. Moreover, 5-CQA was more stable than 3,5-diCQA even at 100 °C. Our results demonstrated that by controlling the cooking temperature and time, new bioactive compounds such as mono- and diCQA derivatives can be produced from sweet potato. These data indicate a potential approach for the development of new functional foods from sweet potato by controlling cooking temperature and time.

## 1. Introduction

Sweet potatoes are nutritionally balanced crops grown in the tropical and temperate regions of the world. Recently, cultivation has increased, especially in African countries. In addition to their excellent nutritional components, sweet potatoes contain many functional components such as yalapin, dietary fiber, and phenols that have been reported to contribute to health [1,2]. In recent years, sweet potatoes have been widely used as a safe and natural food in Japan, and their consumption per capita has been increasing. Accordingly, there is a need to develop better processing methods to obtain functional ingredients.

**Figure 1:**
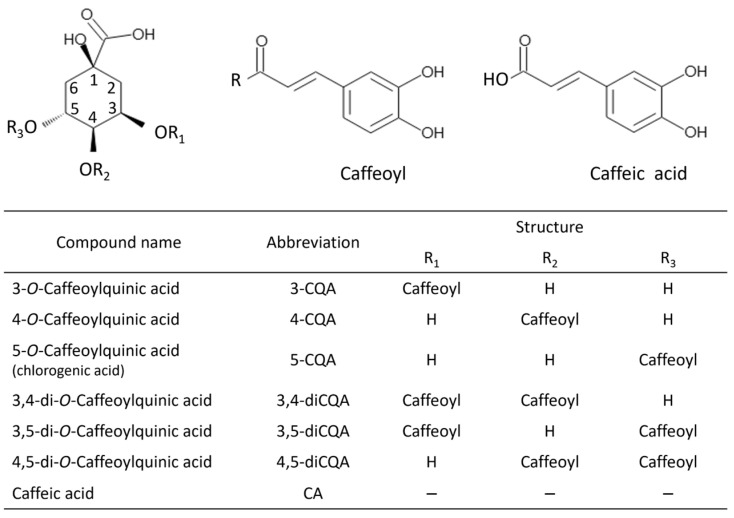
Chemical structures of caffeic acid and monocaffeoylquinic acid (monoCQA) and dicaffeoylquinic acid (diCQA) derivatives. The IUPAC numbering system (IUPAC 1976) [6] is used for these compounds.

The main component of sweet potato phenolic compounds is chlorogenic acid, which belongs to the ester group. This is formed by the condensation of quinic and trans-cinnamic acids such as caffeic acid (CA), p-coumaric acid, and ferulic acid [3]. 5-O-caffeoylquinic acid (CQA) is the most common chlorogenic acid [4]. Takenaka et al. [5] identified seven phenolic compounds using high-performance liquid chromatography (HPLC) and nuclear magnetic resonance (NMR) spectroscopy in the roots of Beniazuma, a variety of sweet potato: CA and six kinds of CQA, namely, 3-CQA, 4-CQA, 5-CQA, 3,4-dicaffeoylquinic acid (diCQA), 3,5-diCQA, and 4,5-diCQA (Figure 1). These are based on the number and position of the caffeoyl groups, and CQAs are classified into various derivatives [6]. Subsequently, HPLC was used to analyze the phenolic composition of root extracts of sweet potato cultivars commonly used in the processing of several traditional Japanese food products [7,8]. CQAs are a broad class of secondary metabolites found in edible and medicinal plants from various families that play an important role in the in vivo defense systems against insects and other pathogens. CQAs have been reported to exhibit antioxidant [9,10], antibacterial [9,10], antiviral [11], cancer-related [12,13,14], antilipidemic [15], antidiabetic [15], antihypertensive [16], anti-Alzheimer [17,18,19], and neuroprotective activity [19,20].

Sweet potatoes are commonly prepared in various ways prior to consumption. Heat treatment improves digestibility [21], induces significant changes in chemical composition, and thus influences the concentration and bioavailability of compounds [22,23]. Heat treatment of sweet potatoes positively influences their chlorogenic acid content, total phenolic content, and antioxidant activity [24]. Shibata et al. [25] reported that the chlorogenic acid in sweet potatoes could be effectively taken up when cooked in a microwave oven or steamed and simmered with cooking water after boiling. However, studies have shown that baking (230 °C), steaming, and microwaving them can decompose phenolic compounds, carotenoids, and flavonoids, as well as vitamin C [26,27,28]. The contents of several phenolic derivatives such as caffeic acid, chlorogenic acid, and vanillic acid decrease during cooking [23]. Changes in these phytochemicals during cooking can also lead to differences in their biological activities. The relationship between the type of cooking method and the final phenolic concentration and antioxidant capacity in sweet potatoes has been the subject of various studies [22,23,24,26,27,28]. However, few studies have examined changes in the CQAs of sweet potatoes during cooking and processing over time. Qualitative and quantitative variations in the bioactive compounds of sweet potatoes may contribute to differences in their health-promoting properties.

To identify whether cooking methods effect the biological activity of sweet potato, in this suty, we first investigated the phenolic contents and 1,1-diphenyl-2-picrylhydrazyl (DPPH) radical scavenging activity during daily cooking methods, such as steaming, deep-frying, and baking.

Furthermore, the structural changes, caused by cooking temperature, to the bioactive compounds, CQAs, were detected using high-performance liquid chromatography (HPLC). Finally, the 5-CQA and 3,5-diCQA standards were heat-treated at 100 °C, and their stability was investigated to determine whether the effect of the sweet potato component was present.

## 2. Materials and Methods

### 2.1. Chemicals

The monoCQAs 3-CQA, 4-CQA, and 5-CQA were purchased from Funakoshi Co., Ltd. (Tokyo, Japan). DiCQA, 3,4-diCQA, 3,5-diCQA, and 4,5-diCQA were purchased from MedChem Express (Monmouth Junction, NJ, USA). The CA (caffeic acid) was purchased from Wako Pure Chemical Industries (Osaka, Japan). HPLC analysis confirmed that the purity of each compound was >97.9%. DPPH was purchased from Tokyo Chemical Industry Co., Ltd. (Tokyo, Japan). All other chemicals, including ethanol, acetonitrile (HPLC grade), and formic acid, were purchased from Nacalai Tesque, Inc. (Kyoto, Japan). 2-(*N*-morpholino) ethanesulfonic acid was purchased from Wako Pure Chemical Industry, Ltd. (Osaka, Japan). All reagents were of analytical grade.

### 2.2. Sample Preparation

“Koganesengan”, a sweet potato variety for Shochu (a distilled alcoholic drink in the Minami Kyushu area in Japan) and for such traditional processed products as deep-fried chips covered with sugar, were purchased from the same farmer in Kagoshima in September (168 ± 21.5 g/raw roots). After being dice-cut into 1 cm pieces and manually peeled using a stainless-steel knife, they were used as raw material in three types of cooking treatments.

### 2.3. Cooking Treatment

Cooking treatments included steaming, deep-frying, and baking. For steaming, the water in a steamer made of stainless steel (26 cm square) was boiled, and the sample was then added and heated for 10, 30, or 60 min. For deep-frying, 800 mL of refined canola oil was placed in a stainless-steel pan (diameter: 18 cm), and the samples were deep-fried at 160 °C for 2, 4, 8, or 16 min. Steaming and deep-frying were performed using a Paloma PA-E18F gas table (Paloma Co., Ltd., Aichi, Japan). For baking, a Rinnai RCK-20AS3 convection oven (Rinnai Co., Ltd., Aichi, Japan) was preheated to 180 °C, and the samples were wrapped in aluminum foil cut into 20 cm squares and baked for 10, 30, or 60 min. After heating, these samples were cooled at room temperature for approximately 1 h and then frozen at −60 °C. Then, these samples were freeze-dried using an Iwaki FRD-50M freeze-dryer (Iwaki Co., Ltd., Tokyo, Japan) and milled using a YDK Y-308B mill (YDK Co., Ltd., Fukushima, Japan) to obtain a uniform powder.

### 2.4. Extraction and Measurement of Total Phenolic Content

Phenolic extracts were prepared as described previously [7]. Briefly, 100 mg of freeze-dried root powder was added to 1 mL of 80% ethanol (*v*/*v*) and boiled for 5 min in an Iwaki RE1-D water bath (Iwaki Glass Co., Ltd., Tokyo, Japan). After centrifugation at 3000 rpm for 15 min using a Kubota 3520 centrifuge (Kubota Corporation Co., Ltd., Osaka, Japan), the supernatant was collected, and the extraction was repeated. The two extracts were combined and made up to 2 mL. The total phenolic content of the extract was measured using the partially modified Folin–Ciocalteu method [29]. Namely, 1.0 mL of 10% Folin-Ciocalteu solution was added to 1.0 mL of a sample solution. After an interval of 3 min, 2.0 mL of 10% sodium carbonate aqueous solution was added. The mixture was allowed to stand for 15 min at ambient temperature, and the absorbance was measured at 600 nm using a PerkinElmer ARVO X3 microplate reader (PerkinElmer Inc, Waltham, MA, USA). The results were expressed as µg of 5-CQA (chlorogenic acid)/100 g dry weight (DW).

### 2.5. Measurement of DPPH Radical Scavenging Activity

DPPH radical scavenging activity was assayed as previously described [30]. Briefly, the extract was diluted with 80% ethanol to obtain various concentrations. Diluted samples (50 μL) were transferred to a 96-well microplate, and 50 μL of 2-morpholinoethanesulfonic acid (MES) buffer (200 mM MES, pH 6.0), 50 μL of 20% ethanol, and 50 μL of DPPH solution (800 μM in ethanol) were added. After allowing the reaction to proceed for 15 min, absorbance was measured at 520 nm using a PerkinElmer ARVO X3 microplate reader (PerkinElmer). Reaction solutions without DPPH were used as color controls, and Trolox was used as the standard. The absorbance at 520 nm (A520 values) was plotted against the diluted sample, and a linear decrease in A520 of the DPPH radical was recorded. A five-point concentration dilution gradient was created to obtain a calibration curve for the DPPH scavenging capacity. DPPH radical scavenging activity was expressed as μM Trolox equivalents (TE)/g of DW.

### 2.6. HPLC Analysis of CQAs

The effects of cooking temperature and heating time of sweet potato on the CQA derivatives were investigated by HPLC according to the retention time (Rt) and UV-vis spectra of the CQA standards [31]. The extract was filtered through a polytetrafluoroethylene membrane (0.20 μm; Merck Biopharma Co., Ltd., Darmstadt, Germany); a 5 μL portion of the filtrate was injected into the HPLC system and then eluted as described below. The HPLC system consisted of a DUG-20A degasser, SIL-20AC auto-injector, CTO-20AC column oven, SPD-M20A diode array detector, CBN-20A system controller, and two LC-10AD pumps (Shimadzu Co., Kyoto, Japan). The system was controlled using an LC solution (version 5.90) workstation (Shimadzu Co., Kyoto, Japan).

A reversed-phase column (75 × 4.6 mm i. d., 3 μm, Cadenza CD-C18: Imtakt, Kyoto, Japan) was used. The mobile phase comprised water containing 0.2% (*v*/*v*) formic acid (A) and acetonitrile (B). The elution was performed using a linear gradient of B as follows: 2% to 19% from 0 to 8 min, 19% to 52% from 8.01 to 16 min, 100% isocratic from 16.01 to 20 min, 100% to 2% from 20 to 20.01 min, and 2% isocratic from 20.01 to 25 min. The column oven was set to 40 °C. The flow rate of B was 1 mL min^−1^. 3-CQA, 4-CQA, 5-CQA, CA, 3,4-diCQA, 3,5-diCQA, and 4,5-diCQA were identified by their retention time and the UV-vis spectra of standards. The quantification of CQAs and CA was performed using external standards based on detection at 326 nm. The peaks of CA and individual CQAs were identified by comparing their peak retention times with those of the reagent standards. Five known concentrations were prepared for each standard and a calibration curve was constructed to determine the concentration of the unknown sample. The results are expressed as g/g DW.

### 2.7. Heating of 5-CQA and 3,5-diCQA

Each 0.25 mg of the 5-CQA and 3,5-diCQA standards was dissolved in 1 mL of 10 mM MES buffer (pH 6.0). These solutions were put in a screw-top glass tube, stoppered, and heated for 10, 30, 60 min in an Iwaki RE1-D water bath (Iwaki Glass Co., Ltd., Tokyo, Japan) set at 100 °C.

### 2.8. Statistical Analysis

All measurements were repeated thrice (*n* = 3). The experimental results were subjected to a one-way analysis of variance (ANOVA) at *p* < 0.05 using Excel Statistics (Bell Curve, Tokyo, Japan), which revealed significant differences. The Tukey–Kramer method was used to identify the significance of the mean differences between the groups. Pearson’s correlation was used to determine the relationship between the total phenolic content and the antioxidant capacity of the sweet potato. The level of significance between groups was set at 5%.

## 3. Results

### 3.1. Weight Change of the Roots after Heating and Freeze-Drying

To investigate the effects of cooking treatments on the total phenolic content and antioxidant capacity, the sweet potato were cooked using three daily cooking methods: steaming, deep-frying, and baking. Each heating time was determined by pretesting to simulate the home cooking method as closely as possible. The roots were used with peels to exploit their functional components. The weights of the roots before and after cooking and after freeze-drying, as well as the percentage fluctuations, are shown in Table 1. The weight of the steamed roots increased, whereas that of the deep-fried and baked roots decreased over time. The order of weights after cooking was steaming > baking > deep-frying. After freeze-drying, the weight of the steamed and baked roots was approximately 36% of the raw weight, whereas that of the fried roots was approximately 39%. Deep-frying absorbed 3% of the oil.

### 3.2. Effects of Cooking on Total Phenolic Content and DPPH Radical Scavenging Activity

**Figure 2:**
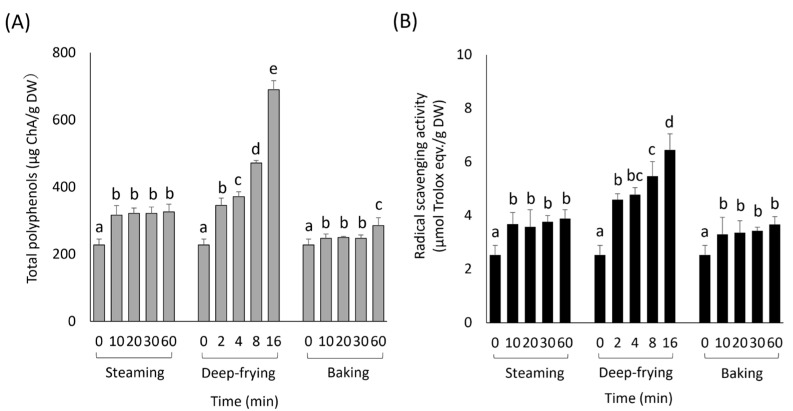
Effects of cooking methods on total phenolic content (**A**) and DPPH radical scavenging activities (**B**). Values are mean ± SD (*n* = 3). Columns with different letters changed significantly for each group (*p* < 0.05).

The effects of cooking on the total phenolic content and DPPH radical scavenging activity are shown in Figure 2. In the steaming treatment, the total phenolic content increased by approximately 1.4 times compared to that of the raw roots at 10 min and remained the same even after 60 min (Figure 2A). Conversely, in the deep-frying treatment, the total phenolic content increased in a time-dependent manner. The contents at 2, 8, and 16 min in the deep-frying treatment increased by approximately 1.5-, 2.1, and 3.0 times compared to the raw roots. In the baking treatment, the total phenolic content increased slightly at 10 min and remained the same up to 30 min. The content increased slightly after 60 min.

The effects of cooking on DPPH radical scavenging activity are shown in Figure 2B. In the steaming treatment, DPPH radical scavenging activities increased by approximately 1.5 times compared to the raw roots at 10 min and remained the same even after 60 min. In contrast, in the deep-frying treatment, DPPH radical scavenging activity increased in a time-dependent manner. The activities increased by approximately 1.8-, 2.2-, and 2.5-fold at 2, 8, and 16 min of deep-frying, respectively, compared to the raw. In the baking treatment, DPPH radical scavenging activities increased by approximately 1.3 times compared to those of the raw roots at 10 min and remained the same until 60 min. The correlation coefficient between the DPPH radical scavenging capacity and total phenolic content was estimated to be 0.976 (*p* < 0.01, *n* = 39).

### 3.3. The Typical HPLC Pattern of CQAs and CA of the Raw Root

**Figure 3:**
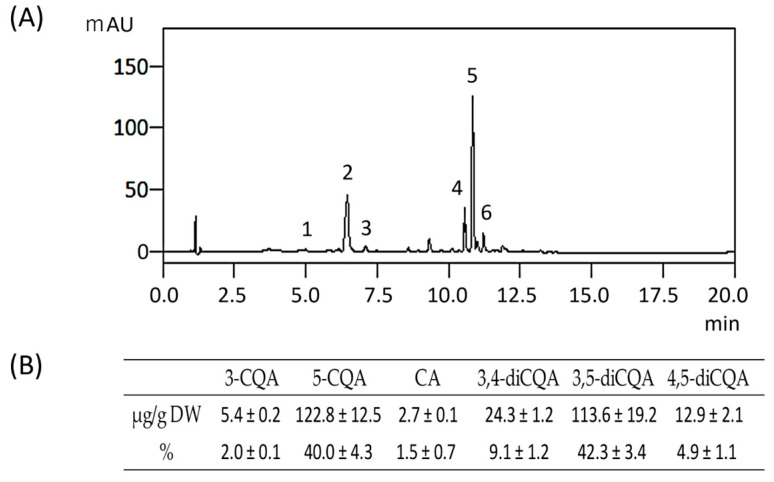
HPLC patterns (**A**) and the content and ratio of each component (**B**) in the raw roots. Peak 1: 3-CQA (Rt = 4.99 min), peak 2: 5-CQA (Rt = 6.44 min), peak 3: CA (Rt = 7.09 min), Peak 4: 3,4-CQA (Rt = 10.55 min), peak 5: 3,5-CQA (Rt = 10.83 min), peak 6: 4,5-CQA (Rt = 11.22 min). Values are mean ± SD (*n* = 3).

The effects of cooking temperature and time on the phenolic content of the sweet potato were investigated using HPLC. A typical HPLC pattern of raw roots is shown in Figure 3. Six peaks, namely, 3-CQA, 5-CQA, CA, 3,4-diCQA, 3,5-diCQA, and 4,5-diCQA, were observed in the raw roots. No peaks corresponding to 4-CQA were observed. The main phenolic components were 5-CQA in the monoCQAs and 3,5-diCQA in the diCQAs. When the total phenolic content of the four types of monoCQAs and three types of diCQAs was 100%, 5-CQA was approximately 40% and 3,5-diCQA was approximately 42%, indicating that 5-CQA and 3,5-diCQA accounted for approximately 82% of the total phenolic content.

### 3.4. Effects of Heating Temperature and Time on monoCQA Derivatives

**Figure 4:**
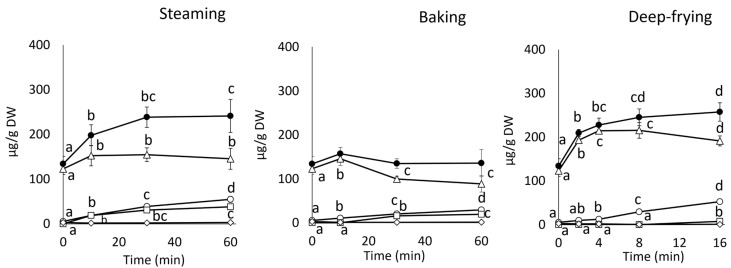
Changes in the contents of monoCQAs and CA in the raw, steamed, deep-fried, and baked roots by treatment times. ○: 3-CQA, □: 4-CQA, △: 5-CQA, ◇: CA, ●: total monoCQAs. The means of the components after the same cooking treatment with different letters changed significantly (*p* < 0.05).

The changes in the monoCQA and CA contents and HPLC patterns after steaming, deep-frying, and baking are shown in Figure 4 and Figure S1. In the steaming treatment, the total monoCQA content increased to approximately 1.5 and 1.7 times compared with the raw roots at 10 and 30 min, followed by a gentle increase. The 5-CQA content increased temporarily at 10 min but changed slightly thereafter. In contrast, the 3-CQA content increased by approximately 3.4 and 10.1 times compared to in the raw roots at 10 and 60 min, respectively. A significant 4-CQA peak was observed after 10 min of steaming, and its content increased with steaming time and was approximately 38 times higher than that of the raw samples at 60 min. The CA content was slightly altered by steaming.

In the baking treatment, the total monoCQA content did not show significant differences. The 5-CQA content increased by approximately 1.2 times that of the raw roots at 10 min, whereas it decreased by approximately 20% and 30% compared with that of the raw roots at 30 and 60 min, respectively. The 3-CQA content increased with baking time and at 60 min was approximately 5.4 times higher than that of the raw roots. The 4-CQA content was not detected at 10 min, although it increased by approximately 16 and 20 times compared to the raw roots at 30 and 60 min, respectively. The baking treatment had no effect on the CA content.

In the deep-frying treatment, the total monoCQA content increased rapidly to approximately 1.6 times compared to the raw roots at 2 min. It then continued to increase slowly, and the content was approximately double that of the raw material after 16 min. The 5-CQA content increased to approximately 1.6 and 1.8 times compared with the raw roots at 2 and 8 min, respectively, after which it gradually decreased. The 3-CQA content increased slightly until 4 min of heating, and then increased by approximately 5.4 and 10 times compared with the raw roots at 8 and 16 min, respectively. The 4-CQA content increased slightly after 16 min, while the CA content remained almost unchanged.

### 3.5. Effects of Heating Temperature and Time on diCQA Derivatives

**Figure 5:**
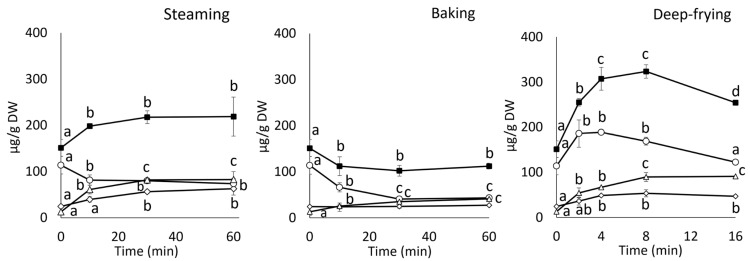
Changes in the contents of diCQAs in the raw, steamed, deep-fried, and baked roots by treatment times. ◇: 3,4-CQA, ○: 3,5-CQA, △: 4,5-CQA, ■: total diCQAs. The means of the components after the same cooking treatment with different letters changed significantly (*p* < 0.05).

The changes in the diCQA content and HPLC patterns after steaming, deep-frying, and baking are shown in Figure 5 and Figure S2.

In the steaming treatment, the total diCQA content increased by approximately 1.3 times compared to the raw roots at 10 min and changed little thereafter. The 3,5-diCQA content decreased by approximately 28% compared to that of the raw roots at 10 min; thereafter, the heat treatment did not influence it. In contrast, the 3,4-diCQA content increased approximately 2.3 times higher than that in the raw roots at 30 min and then slightly increased. The 4,5-diCQA content increased to approximately 4.7 and 6.3 times as compared to the raw roots at 10 and 30 min, and it changed slowly thereafter.

In the baking treatment, the total diCQA content was reduced by 25% of the raw diCQA content at 10 min, and then changed little. The 3,5-diCQA content decreased dramatically with increasing baking time. It decreased by 42% and 62% compared to the raw roots at 10 and 60 min, respectively. In contrast, the 3,4-diCQA content did not change after baking. The 4,5-diCQA content was increased to approximately 2.0 and 3.2 times as compared to the raw roots at 10 and 60 min, respectively.

In the deep-frying treatment, the total diCQA content increased rapidly to approximately 1.7 times compared to the raw roots at 2 min. After reaching approximately 2.1 times at 8 min, and after 16 min, it reduced to 30% of the 8 min level. The 3,5-diCQA content increased by approximately 1.6 times at 2 min, whereas it decreased from 4 min. After 16 min, it was reduced by 35% of the 4 min level and was at the same level as that of the raw samples. In contrast, the 3,4-diCQA content increased slowly and was approximately 2.0 times higher than that in the raw roots at 4 min. Thereafter, it remained unchanged. The 4,5-diCQA content increased to approximately 4.7 and 6.3 times as compared to the raw roots at 2 min and 8 min, and it changed slowly thereafter.

### 3.6. Effects of Heating Time on 5-CQA and 3,5-diCQA Standards at 100 °C

**Figure 6:**
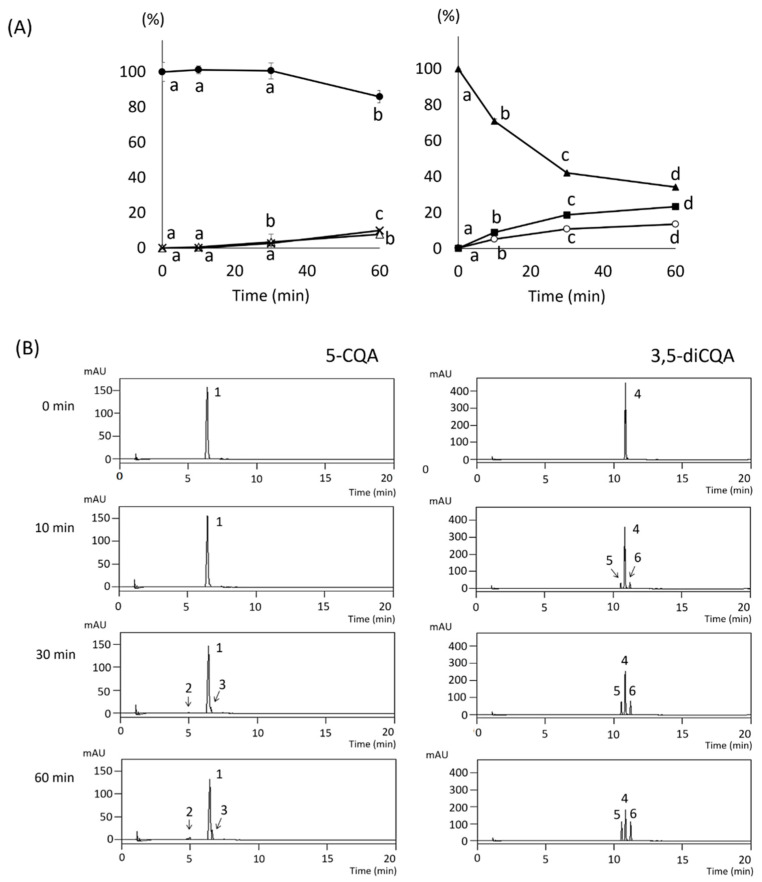
Changes in the contents and HPLC patterns of 5-CQA and 3,5-diCQA standards by heating at 100 °C. (**A**) Relative contents: △: 3-CQA, ×: 4-CQA, ●: 5-CQA, ○: 3,4-CQA, ▲: 3,5-CQA, ■: 4,5-CQA. The means of the components after the same cooking treatment with different letters changed significantly (*p* < 0.05). (**B**) HPLC patterns: peak 1: 5-CQA, peak 2: 3-CQA, peak 3: 4-CQA, peak 4: 3,5-diCQA, peak 5: 3,4-diCQA, peak 6: 4,5-diCQA.

The cooking treatment had a more pronounced effect on the main components of the raw root, 5-CQA and 3,5-diCQA. Therefore, the thermal stability of 5-CQA and 3,5-diCQA standards were investigated at 100°C (Figure 6). The heating times were 0, 10, 30, and 60 min. Heating the standard preparations of 5-CQA at 100°C showed little change until 30 min, and it had decreased by 14% compared to before heating at 60 min. The 3-CQA and 4-CQA levels increased slightly at 30 min, and at 60 min they increased by approximately 2 and 4 times compared with those at 30 min. The cooking methods, including steaming, deep-frying, and baking, as well as the phenolic contents and DPPH radical scavenging activities and structural changes in the CQAs were investigated. First, the phenolic content and DPPH radical scavenging activity were examined. The results showed that the 3,5-diCQA content decreased with increasing heating time. It decreased by approximately 30, 58%, and 66% compared to that before heating at 10, 30, and 60 min, respectively. In contrast, 3,4-diCQA and 4,5-diCQA contents increased with increasing heating time.

## 4. Discussion

### 4.1. Effect of Cooking Methods on Phenolic Components

The purpose of this study was to clarify the effects of cooking sweet potatoes on their polyphenols and antioxidant capacity using the usual cooking methods of steaming, deep-frying, and baking at home. The main phenolic components in the raw sweet potatoes were 5-CQA and 3,5-diCQA (Figure 3).

5-CQA and 3,5-diCQA accounted for approximately 40% and 42% of the total phenolics in this study, respectively, representing approximately 82% of the phenolic content of raw sweet potatoes. These results agree with those of previous reports [3,6,17,23,27]. The antioxidant capacity of heated roots was suggested to be highly related to the phenolic-derived compounds that were affected by the cooking process.

Changes in phytochemicals upon cooking may result from two opposing phenomena: a matrix softening effect, which increases the extractability of phytochemicals, resulting in a higher concentration with respect to the raw material, and thermal degradation, which reduces their concentration [21]. Therefore, the extraction rates of 3,5-diCQA and 5-CQA increased immediately after cooking, but 3,5-diCQA in the steaming and baking treatments may have also undergone degradation.

Previous reports indicate that 5-CQA and 3,5-diCQA are unstable at high temperatures, and heating in the temperature range of 100–200 °C results in isomerization and other transformations [32,33]. Isomerization was promoted by the high water content of the sample [34]. The total content of these monoCQAs remained at almost the same level as that in deep-frying, with the total diCQA content being approximately 30% lower, suggesting the isomerization of both the 5-CQA and 3,5-diCQA components due to the high moisture content during heating in the steaming treatment (Table 1).

Derivatives were produced in the baking treatment, but in small quantities, and the total amounts of monoCQAs and diCQAs were also lower than those produced by the other cooking methods. Carrera et al. reported that roasting at 240 °C for 30 min increased the levels of CQA derivatives in sweet potatoes [35]. Murakami et al. [36] found that aqueous chlorogenic acid solutions are more readily decomposed at 180 °C than at 100 °C, suggesting a role for chlorogenic acid in protecting rutin coexisting in foods from thermal decomposition. Shibata et el. reported that 5-CQA and 3,5-diCQA were thermally decomposed during a baking treatment at 230 °C for 25 min [25]. In our results, the low baking temperature (180 °C) may be the reason why the quantities of mono- and diCQAs were low.

The thermal decomposition of CQAs occurs more quickly at higher temperatures, as evidenced by coffee roasting [37,38]. The isomerization of diCQA was more aggressive than that of monoCQA during deep-frying. The heating temperature was 160 °C and the heating time was short in the deep-frying, which probably resulted in fewer 4-CQA derivatives (Figure 4 and Figure S1). These changes in the CQAs indicate that isomerization and decomposition occurred depending on the heating temperature. 3,5-diCQA was also shown to be more thermally unstable than 5-CQA.

Raw sweet potato were confirmed to contain approximately 82% 5-CQA and 3,5diCQA, which were isomerized by heating to produce monoCQA and diCQA derivatives, respectively. It has been reported that 3,5-diCQA prevents neuronal apoptosis by protecting mitochondrial activity [39]. In addition, 3,4-diCQA and 3,5-diCQA in Brazilian propolis have been found to contribute to its antihypertensive effects [40]. The α-glucosidase inhibitory activities of diCQA derivatives are reported as follows: 1,4-diCQA > 1,5-diCQA > 3,4-diCQA > 4,5-diCQA > 3,5-diCQA. Moreover, the higher glucose-stimulated insulin secretory activity of diCQA derivatives has been reported as follows: 4,5-DCQA > 3,4-DCQA > 1,4-diCQA > 3,5-diCQA > 1,5-diCQA [41]. The reports indicated that the 3,4-diCQA, 4,5-diCQA, and 3,5-diCQA derivatives were more functional than 3,5-CQA. This study showed that sweet potato contained high amounts of diCQA derivatives after cooking, indicating the potential of both monoCQA and diCQA derivatives as functional foods. Controlling the heating temperature and time may enable the production of processed products with distinct functional characteristics.

### 4.2. Effect of Cooking Methods on DPPH Radical Scavenging Activity

The phenolic content and antioxidant capacities were the highest (Figure 2), despite the low total amount of CQAs, at 16 min in the deep-frying treatment (Figure 4 and Figure 5). The 50% radical scavenging concentration (SC50) of 5-CQA isolated from mulberry leaves was reported as 32.76 ± 0.27 μg/mL, while that of 4-CQA was 11.41 ± 0.48 μg/mL. The free radical scavenging activity of 4-CQA is approximately three times higher than that of 5-CQA [42]. Jiang et al. reported that the free radical scavenging activities of each compound, especially tri-CQA and diCQAs, were determined experimentally and found to be significant [43]. In addition, the number of caffeoyl groups in caffeoyl derivatives plays a crucial role in these physiological functions [43]. Thus, our data support that 5-CQA and 3,5-diCQA contributed to the antioxidant capacity before heating, and their isomerized derivatives, such as 3- and 4-CQA as well as 3,4- and 4,5-diCQA, contributed to the antioxidant capacity of sweet potatoes after cooking and heating. However, the amount of each derivative and the total CQA content in the baking treatment were lower than those in the other treatments. Heating causes polymerization and oxidative degradation of phenolic compounds, producing browning substances composed of a complex group of compounds with antioxidant capacities [44]. Murakami et al. [36] reported that even though phenolics were rapidly decomposed by heating at 180 °C, some degradation products still exhibited radical scavenging activity. Moreover, cooked sweet potatoes contain not only phenolics but also other substances, such as maltose and other sugars, amino acids, and vitamins, which cause Maillard reactions and caramelization depending on the heating temperature and time [45,46]. Substances produced by these reactions exhibit antioxidant properties [45]. These reactions suggest that they may also have contributed to the increased antioxidant capacity in the deep-frying treatment and the retention of antioxidant capacity in the baking treatment. Therefore, the heating temperature and time of the roots should be controlled during cooking to increase antioxidant capacity.

These results indicated that 5-CQA and 3,5-diCQA, the main phenolic compounds in the raw root, were isomerized and degraded by heating. The effects of cooking on the phytochemical content depend on the processing conditions, the structure of the food matrix, and the chemical nature of the specific compound. The stability of CQAs is reportedly improved by the addition of epigallocatechin gallate and vitamin C [47]. Therefore, each standard was heated at 100 °C for a certain period, and its heat resistance was examined (Figure 6). The pH was set to 6.0, which was the same as that of the root condition.

5-CQA produced tiny amounts of 3-CQA and 4-CQA derivatives after heating at 100 °C, and it was relatively stable. In contrast, 3,5-diCQA decreased more rapidly upon heating the raw roots, and 3,4- and 4,5-diCQA derivatives were produced. 3,5-diCQA was shown to undergo isomerization and degradation over time with increasing heating times. However, monoCQA derivatives that were expected from the degradation of 3,5-diCQA were not observed until heating at 100 °C for 60 min.

3,5-diCQA was shown to be more thermally unstable than 5-CQA. However, the effect of temperature on each sweet potato component requires further investigation.

Beverages, especially coffee, contribute a large share of the consumption of phenolics as antioxidants in the Japanese diet [48]. The concentration of total phenolics in coffee is reported to be 200 mg/100 mL [48]. Unroasted coffee beans are rich in CQAs, but roasting induces isomerization, with a decline in 5-CQA and a concomitant increase in 3-CQA and 4-CQA. There are very few diCQA derivatives in roasted coffee beans compared to monoCQA derivatives [37]. It has been reported that 5-CQAs are the major contributors to the antioxidant capacity of green coffee beans, whereas after roasting, 5-CQA decreases and 3-CQA and 4-CQA contribute to the antioxidant activity [38].

## 5. Conclusions

The daily cooking methods of steaming, deep-frying, and baking significantly enhanced the phenolic content of sweet potato, with a positive correlation with DPPH radical scavenging activity. In particular, the deep-frying treatment resulted in higher antioxidant capacity with increasing heating time. The major components, 5-caffeoylquinic acid and 3,5-dicaffeoylquinic acid, present in raw sweet potatoes were isomerized to bioactive 3- and 4-caffeoylquinic acid as well as 3,4- and 4,5-dicaffeoylquinic acid, respectively. Therefore, the daily cooking of sweet potatoes by controlling the heating temperature and time can produce new functional foods from sweet potato.

## Figures and Tables

**Table 1:** Weight and fluctuation before and after heating and after freeze-drying.

Steaming						Baking						Deep-Frying					
Time (min)	Before Heating (g)	After Heating (g)	Fluctuation A (%)	DW (g)	Fluctuation B (%)	Time (min)	Before Heating (g)	After Heating (g)	Fluctuation A (%)	DW (g)	Fluctuation B (%)	Time (min)	Before Heating (g)	After Heating (g)	Fluctuation A (%)	DW (g)	Fluctuation B (%)
0	30.6 ± 4.6			11.1 ± 1.6	36.3 ± 3.3	0	30.6 ± 4.6			11.1 ± 1.6	36.3 ± 3.3	0	30.6 ± 4.6			11.1 ± 1.6	36.3 ± 3.3
10	33.9 ± 6.6	34.6 ± 6.7	102.0 ± 0.3	12.4 ± 2.4	36.5 ± 2.1	10	39.6 ± 9.9	37.8 ± 9.6	95.5 ± 0.7	14.5 ± 4.1	36.4 ± 2.4	2	44.4 ± 6.3	33.8 ± 5.0	76.1 ± 1.9	17.6 ± 2.6	39.5 ± 1.2
30	45.7 ± 9.1	46.4 ± 9.1	101.5 ± 0.6	16.8 ± 3.5	36.7 ± 1.8	30	48.2 ± 6.1	41.0 ± 5.7	84.9 ± 1.3	17.6 ± 2.7	36.4 ± 2.0	4	41.9 ± 2.7	27.8 ± 2.2	66.3 ± 1.5	16.6 ± 1.0	39.7 ± 1.1
60	45.2 ± 12.1	45.9 ± 12.3	101.5 ± 0.8	16.7 ± 4.4	37.0 ± 2.5	60	47.2 ± 10.5	34.3 ± 9.5	72.0 ± 3.6	17.5 ± 4.0	36.9 ± 2.2	8	39.3 ± 4.9	22.8 ± 2.9	58.1 ± 2.2	15.9 ± 2.3	40.3 ± 1.8
												16	37.7 ± 6.8	18.5 ± 2.4	49.7 ± 4.1	15.2 ± 1.9	40.9 ± 5.1

Values are mean ± SD (*n* = 3). Fluctuation A = after heating (g)/before heating (g) × 100; fluctuation B = dry weight (DW) (g)/before heating (g) × 100.

## Data Availability

The original contributions presented in the study are included in the article and Supplementary Material, further inquiries can be directed to the corresponding author.

## Supplementary Materials

The following supporting information can be downloaded at: https://www.mdpi.com/article/10.3390/foods13071101/s1, Figure S1: HPLC patterns of monoCQAs and CA in the raw, steaming, deep-frying, and baking treatment roots. Peak 1: 3-CQA, peak 2: 5-CQA, Peak 3: 4-CQA, peak 4: CA; Figure S2: HPLC patterns of diCQAs in the raw, the steaming, deep-frying, and baking treatment roots. Peak 1: 3,4-CQA, peak 2: 3,5-CQA, peak 3: 4,5-CQA.
