# Supplementary material for: Effects of Cooking Methods on Caffeoylquinic Acids and Radical Scavenging Activity of Sweet Potato
Source: Foods. 2024 Apr 3;13(7):1101. doi: 10.3390/foods13071101 (PMC11011517; doi:10.3390/foods13071101)
Supplement: Supplementary file 1 [file foods-13-01101-s001.zip › foods-2919611-supplementary.pdf]

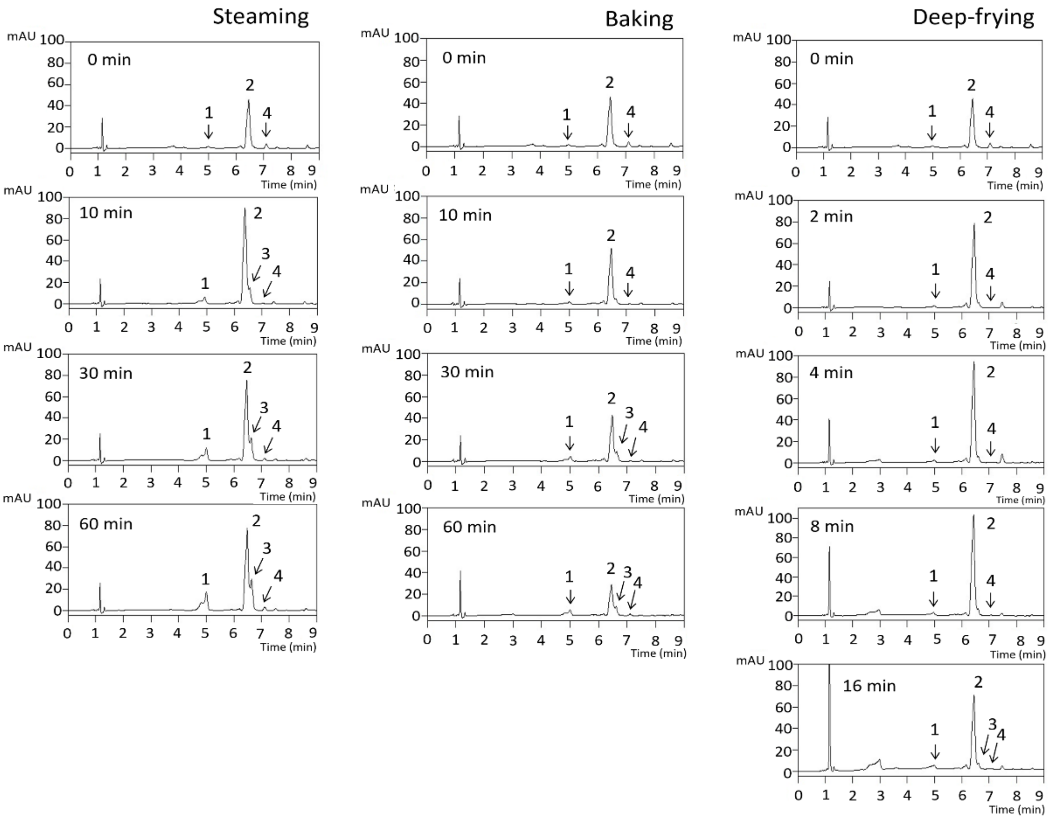


Figure S1 (Supplementary materials). HPLC patterns of monoCQAs and CA in the raw, steaming, deep-frying, and baking treatment roots. Peak 1: 3-CQA, peak 2: 5-CQA, Peak 3: 4-CQA, peak 4: CA.

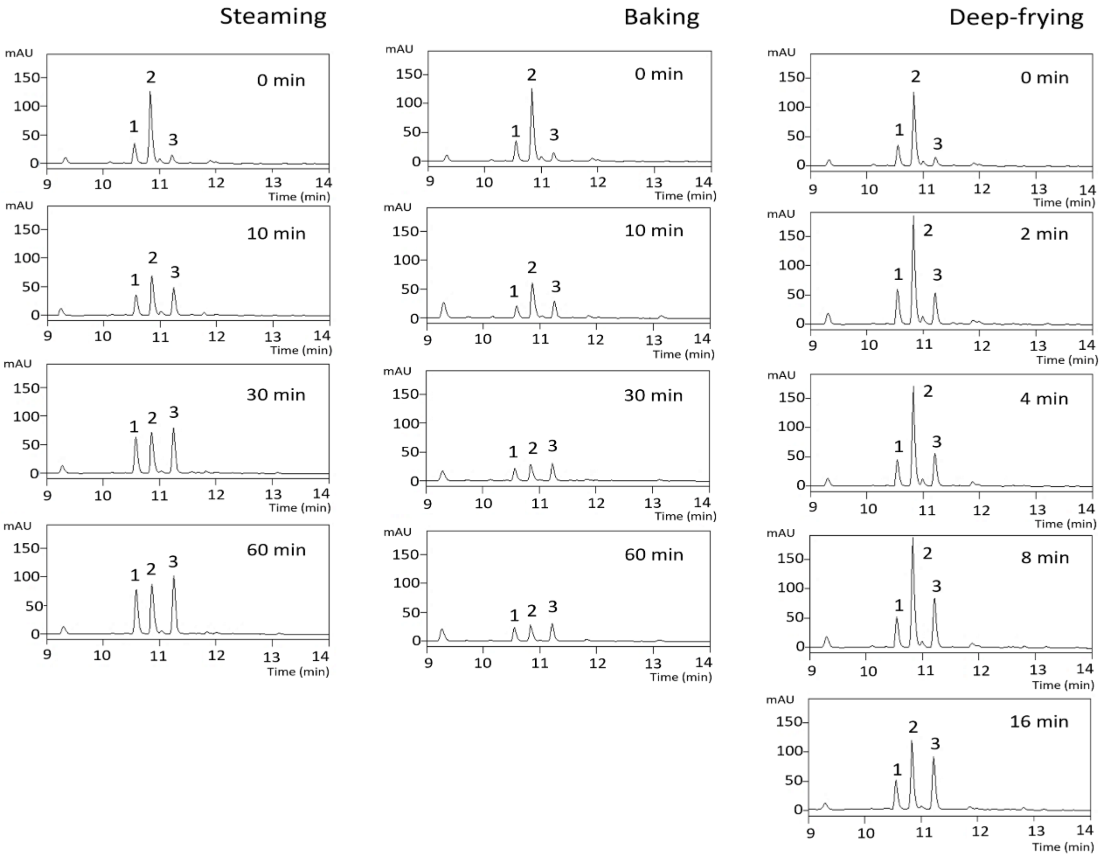


Figure S2 (Supplementary materials). HPLC patterns of diCQAs in the raw, the steaming, deep-frying, and baking treatment roots. Peak 1: 3,4-CQA, peak 2: 3,5-CQA, peak 3: 4,5-CQA.
